# Hybrid Beamforming in Massive MIMO for Next-Generation Communication Technology

**DOI:** 10.3390/s23167294

**Published:** 2023-08-21

**Authors:** Shahid Hamid, Shakti Raj Chopra, Akhil Gupta, Sudeep Tanwar, Bogdan Cristian Florea, Dragos Daniel Taralunga, Osama Alfarraj, Ahmed M. Shehata

**Affiliations:** 1 School of Electronics and Electrical Engineering, Lovely Professional University, Phagwara 144411, India; shahid.12100240@lpu.in (S.H.); akhil.20239@lpu.co.in (A.G.); 2 Department of Computer Science and Engineering, Institute of Technology, Nirma University, Ahmedabad 382481, India; sudeep.tanwar@nirmauni.ac.in; 3 Department of Applied Electronics and Information Engineering, Faculty of Electronics, Telecommunications, and Information Technology, National University of Science and Technology Politehnica, 061071 Bucharest, Romania; 4 Computer Science Department, Community College, King Saud University, Riyadh 11437, Saudi Arabia; oalfarraj@ksu.edu.sa; 5 Computer Science and Engineering Department, Faculty of Electronic Engineering, Menoufia University, Menofia 32511, Egypt; am.shehata@el-eng.menofia.edu.eg

**Keywords:** MIMO, massive MIMO, beamforming, hybrid beamforming

## Abstract

Hybrid beamforming is a viable method for lowering the complexity and expense of massive multiple-input multiple-output systems while achieving high data rates on track with digital beamforming. To this end, the purpose of the research reported in this paper is to assess the effectiveness of the three architectural beamforming techniques (Analog, Digital, and Hybrid beamforming) in massive multiple-input multiple-output systems, especially hybrid beamforming. In hybrid beamforming, the antennas are connected to a single radio frequency chain, unlike digital beamforming, where each antenna has a separate radio frequency chain. The beam formation toward a particular angle depends on the channel state information. Further, massive multiple-input multiple-output is discussed in detail along with the performance parameters like bit error rate, signal-to-noise ratio, achievable sum rate, power consumption in massive multiple-input multiple-output, and energy efficiency. Finally, a comparison has been established between the three beamforming techniques.

## 1. Introduction

Cellular technology evolved from 1G in the early 1980s to 2G, which includes GSM, in the early 1990s. After this, 2.5G was developed, followed by 3G (2001), which uses the technique of both circuit and packet switching, hence, achieving data rates of 144 kbps and 2 Mbps, respectively. Both 3.5G and 4G use the technique of packet switching only and achieve relatively higher data rates, up to 100 Mbps, to boost capacity and spectrum efficiency in 4G, multiple-input multiple-outputs (MIMO) technology is used as presented by K. Santhi et al. in [1], for example, 8 × 8 multiple-input multiple-outputs (MIMO). In legacy LTE, the term MIMO primarily refers to single-user MIMO (SU-MIMO), where multiple data streams are given to the UE at once, utilizing the same time/frequency resources in single-user MIMO and, thus, increasing the link data rate. The second type of MIMO is called multi-user MIMO (MU-MIMO), where multiple data streams are focused on multi-users. MU-MIMO increases the efficiency of the spectrum. There are tens or hundreds of antennas employed in the MU-MIMO system. The channel estimation accuracy in MU-MIMO is dependent upon the channel state information (CSI). The number of new implementations has grown, which has raised user demand. The fifth generation (5G) wireless network is now taking off, promising to provide exceptional customer satisfaction and open the door to a wide range of informational content. In this way, 5G is paving the way for fully digitizing and linking the world. As a result, telecom networks are conducting research into key 5G and beyond technologies for essential certification and confirmation, as stated by the authors of [2] in their study, where they outlined the requirements for a 5G network, such as high-frequency spectra with a wide bandwidth, base station densification, and a significant number of antennas to facilitate efficient data transfer [3]. Reduced device power consumption, path loss, better coverage area, greater data rates, increased spectral efficiency, and interference suppressing are a few of the design considerations in 5G. To achieve these requirements, various algorithms and methodologies have been designed and are being used, for example, the cutting-edge access techniques Beam Division Multiple Access (BDMA), which is the technique where a broader beam is divided into multiple narrower beams via user scheduling and Non- and Quasi-Orthogonal or Filter Bank Multicarrier (FBMC) Multiple Access and many other hardware architectures for practical implementation, along with the above-mentioned technologies, massive MIMO has a primary position in techniques used to achieve the above-mentioned requirements. Massive MIMO is a technique where the base station is employed with hundreds of antennas which, in turn, employs the beamforming. By precisely balancing the magnitude and phase of each antenna signal, beamforming uses several antennas to control the direction of wavefronts. Some of the beamforming technologies and their contributions are briefly mentioned in Table 1.

The very near future of wireless technology is 6G, with some important characteristics [14] presented in Table 2.

## 2. Beamforming

**Figure 1:**
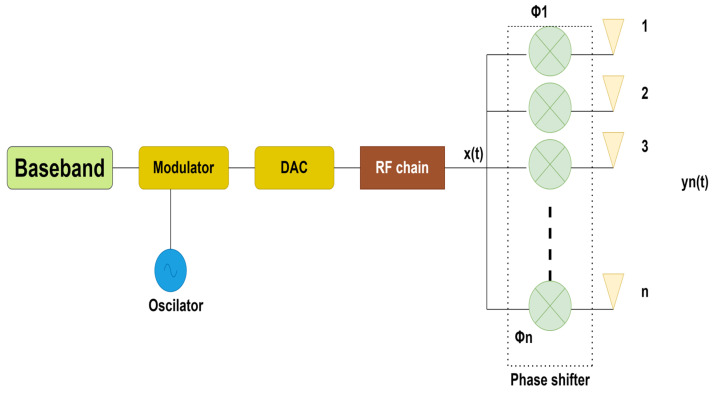
Analog beamforming.

In massive MIMO, each antenna element is controllable, unlike the conventional array and full dimensional MIMO (FD-MIMO); this controllable character will enable beamforming, which is the soul of 5G and future communication technology, and in this process, radio waves will be concentrated in a narrow angular region which will improve spectral efficiency significantly [4]. Beamforming involves adjusting the magnitude and period of each data signal transmitted from multiple antennas to steer the signal in a specific spatial direction, leveraging the channel state information (CSI) convenient at the sending end. Precoding/beamforming is primarily used to improve the network effectuation by exploiting the CSI. More antennas mean the beam will be narrower, intended to overcome the shortcomings of interference mitigation strategies that cannot successfully battle inter-user interference (IUI) [15]. Beamforming is a process in which a radiation pattern is directed toward a particular region only, creating constructive interference in certain desired angles and destructive interference in undesired angles [16]. It was first demonstrated in 1905 by German scientist Karl B. Braun by developing a phased array of three antenna elements [17]. The more antennas involved in beamforming, the greater the directionality of the beam, and more power will be directed to the user equipment [18], and the spatial power distribution will be given by the vector sum of fields emitted by the individual antenna elements (also termed as array radiation pattern). Beamforming is also accepted in different fields like Sound Navigation And Ranging (SONAR), Radio Detection And Ranging (RADAR), Acoustics, Seismology, Biomedical, and Wireless Communications. It enhances the function of a communication system by employing the interference rescission concept while using efficient beamforming algorithms. Signal intensity and degree of departure can be altered via beamforming, which also aids in voltage modulation and prudent beam guiding; therefore, beamforming security is increased, as the signal will only be received by the intended receiver [19]. The primary achievement of beamforming is the transmit/receive gain when compared to omnidirectional transmission. Beamforming is divided into three architectural categories: analog, digital, and hybrid. Before moving on to hybrid beamforming, let us first quickly discuss analog and digital beamforming. The simplest method is analog beamforming, which uses a single-phase adjustment in the analog domain. With analog beamforming, the output of a single radio frequency (RF) transceiver is divided into several routes that correspond to the array’s antennas. Each signal is put through a phase shifter before getting to the antenna element. Each transmitting component has a phase and amplitude controller that pedals the transmitted signals’ phase and amplitude, respectively. Active beamforming antennas also enable the transmitting element to contain a power amplifier, in contrast to passive beamforming antennas, which only include a single high-power amplifier in the RF chain. An analog beam former can only form one beam at a time since it has a single RF chain. Due to minimum hardware and software overhead and minimal energy usage of phase shifters and dampers, this beamforming approach is the simplest, most affordable, and energy-efficient, as shown in Figure 1. It performs significantly less due to lower antenna gain [20,21].

**Figure 2:**
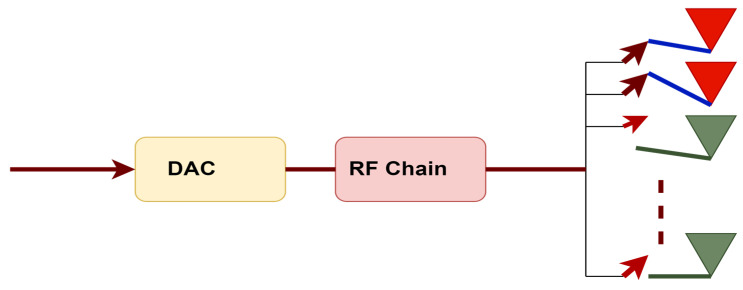
On–Off analog beamforming.

On–Off analog beamforming (OABF) is another analog beamforming system with low complexity. Other antenna elements are detached, and only a subset of the antennas that do not utilize analog signal processing is coupled to the RF chain to transmit data. RF switches are employed for turning one antenna element on and others off. This is why it is called On–Off analog beamforming. Figure 2 displays a block representation of OABF, assuming two antenna elements are connected and others open-circuited. The idea behind creating OABF was to choose a subset of antennas with favorable working conditions and matching phases [5].

**Figure 3:**
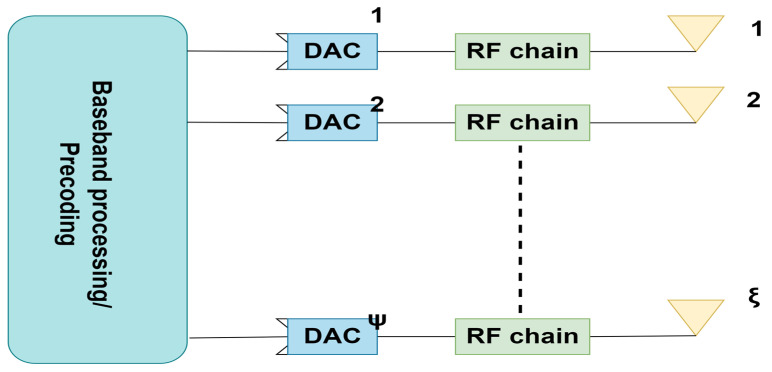
Digital beamforming.

The second beamforming technology discussed in this article is digital beamforming. It can be described as the technology where each antenna requires a separate RF chain. The advantages of digital beamforming over analog beamforming include its support for spatial multiplexing, which enables different directivity for various frequency subcarriers, and its ability to detect and eliminate interference by adjusting the period and magnitude of the original bit stream. The baseband is where all signal processing is performed. A digital baseband precoder individually controls each antenna element’s amplitude and phase of the broadcast signals [6], as in Figure 3, where we assumed ψ number of DACs and ξ number of antenna elements. Based on the angle of arrival (AOA) from the transmitters, there are two different methods of digital beamforming. The ideal array weights needed to drive the antenna array elements to achieve the desired beamforming pattern do not need to be changed if the angles of arrival do not change over time. This type of beamforming is called fixed. The ideal array weights must be adjusted if the wireless channel changes over time. Real-time computation of the ideal array weights is required. This type of digital beamforming is called adaptive beamforming [22]. Various linear precoding techniques limit inter-user interference (IUI), such as zero-forcing (ZF), regularized zero-forcing (RZF), maximum ratio transmission (MRT), etc.

*Maximum Ratio Transmission (MRT)* is a precoding method that uses the Hermitian of the channel matrix to optimize the SINR at the prearranged recipient [23]. To accomplish this, the base station uses the MRT precoding matrix, which is given as follows.

(1) $$W=H^{H},$$

where *W* is the precoding matrix and MRT is preferred because it does not need channel inversion and is the simplest of precoding techniques.

*Zero-Forcing (ZF)* is a spatial data processing technique in wireless communication systems with multiple transmitting antennas. Its primary objective is to eliminate the interference between multiple users by applying a precoding matrix that cancels out the interference signals. The precoding matrix is designed to make the channel matrix invertible, which is achieved by ensuring that the inverse of the channel matrix is equal to the product of the precoding matrix and the original channel matrix [24] and is given as follows.

(2) $$W=H^{H}{\left(HH^{H}\right)}^{-1},$$

Equation (2) can act as the left inverse of *H*, therefore, also called the pseudo-inverse of *H*.

*Regularized Zero-Forcing (RZF)* is a precoding technique employed in communication networks as an alternative to directly inverting the channel matrix in zero-forcing (ZF) precoding. RZF precoding uses regularized channel inversion, which involves adding a regularization term to the channel matrix’s inverse to improve the system’s performance. The regularization term helps to balance the noise suppression and signal alignment requirements, resulting in better performance than traditional ZF precoding, and is mathematically represented as follows.

(3) $$W=B{\left(HH^{H}+\alpha I_{k}\right)}^{-1}H^{H},$$

where α gives the regularization factor that needs to be optimized and B represents the power normalization parameter and $I_{k}$ stands for identity matrix.

The comparative description of analog and digital beamforming is depicted in Table 3, where ↑ denotes high and ↓ denotes low.

## 3. MIMO and Massive MIMO

**Figure 4:**
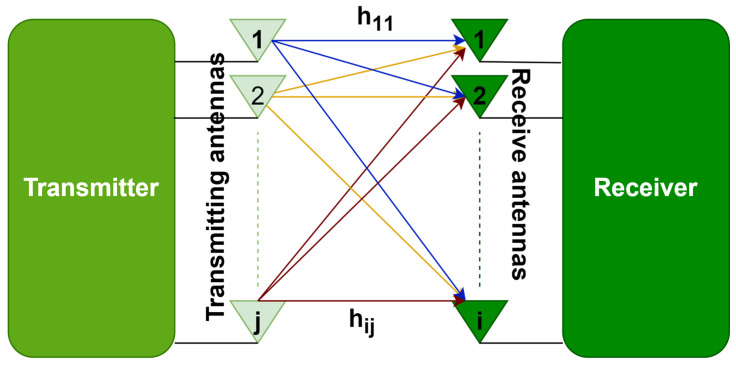
MIMO.

The proliferation of wireless data traffic across communication networks exerts peer pressure on the system. In wireless systems, communication is vulnerable to hazards of manipulation when there is just one route between the sender and the recipient. MIMO technology allows the creation of several links between the transmitting and receiving ends, as shown in Figure 4, thus, increasing communication efficiency. This employment of multiple antenna elements at the transmitting and receiving ends enables data to travel simultaneously through several signal channels, minimizing mistakes, accelerating information speed, and expanding the potential of communication systems.

A favorable propagation condition is created when the BS deploys many antennas, making the wireless channel nearly predictable because the BS-to-UE wireless links are nearly orthogonal to one another. The MIMO approach has uncovered a broad spectrum of applications in numerous fields, including enforcement agencies, network television broadcasting, and the defense industry. The breakthrough in antenna technologies with different settings, such as multiple-input single-output (MISO) and single-input multiple-outputs (SIMO), paved the way for developing MIMO technology that supports more antennas, networks, and devices. The wireless industry constantly explores innovative ways to improve and expand MIMO technology. For instance, massive 5G MIMO systems have been introduced to accommodate more users and increase bandwidth. The MIMO systems can be configured in different ways, such as 2 × 2, 4 × 4, 6 × 6, and 8 × 8, which large 5G systems can customize to provide high network capacity. Moreover, there are two main subcategories of MIMO systems: single-user (SU) and multi-user (MU). SU-MIMO devices can only communicate with one network element instantaneously, while MU-MIMO devices can transmit multiple data symbols simultaneously to multiple devices, rendering them more efficient than SU-MIMO systems.

*Why Massive MIMO* It is a technique that employs an enormous number of independently programmable antenna elements (e.g., 256 antennas at the base station [13]), usually on the base station end of the wireless communication link, to adaptively alter the elevations and azimuth angles of the signal propagation [25,26]. Furthermore, performance and the signal-to-interference-plus-noise ratio are enhanced. The potential paybacks of the massive MIMO are as follows.

- *Potentiality and link reliability* Diversity gain increases in massive MIMO, improving connection stability by preventing fading [27]. Additionally, it is acknowledged that potential/capacity grows with increasing the antenna number. In [28], it has been demonstrated that the potential is increased with growth in the count of antenna elements. A MIMO system can create multiple parallel subchannels in space through spatial multiplexing, increasing channel capacity. The total potential of the structure is determined by the sum of all subchannels, which can be significantly improved through the MIMO technique. For this reason, large-scale MIMO technology is being adopted in 5G systems.

(4) $$C=E\left\{\log_{2}\left[det\left(I_{nr}+\frac{E_{s}}{n_{t}\sigma^{2}}HH^{*}\right)\right]\right\},$$

- *Spectral efficiency* By expanding the multiplicity of spatial streaming data, massive MIMO provides improved spectrum performance, throughput, and multiplexing gain [29].

(5) $$T_{h}=B$$

where $T_{h}$ is the throughput, B is the bandwidth, and SINR is the signal-to-interference-plus-noise ratio. Assuming more separate routes than a standard MIMO system, a massive MIMO that has an exceptionally high number of transmit antennas might potentially even attain a greater system performance.

- *Energy efficiency* The proportion of the network throughput (estimated in bits per second) to the system’s overall use of power (estimated in watts) is known as energy efficiency (EE) in MIMO systems.

(6) $$EE=\frac{T}{W}$$

where *T* is system throughput and *W* is the total power consumption of the setup.

To attain a sufficient level of performance, a MIMO system’s efficiency in using its power resources is measured by EE. A stronger EE suggests improved power management and use, whereas a reduced EE shows inefficiencies. Adaptive modulation and coding, power distribution, and beamforming are a few examples of approaches that can be used to maximize EE in MIMO setups. According to [30], the transmitted power (p_t_) and number of antennas (n_t_) are inversely related as a result of coherent combining. The transmitted power will decrease considerably as n_t_ rises; hence, power per antenna should be inversely proportional to n_t_. In [31], the authors kept the transmitted power constant, and it is demonstrated that throughput is exactly proportional to the quantity of transmitting antennas. The authors in [32] describe the minimal power usage (in milliwatts), concluding that energy efficiency improves with an increase in the number of antennas at the transmitter and receiver.

- *Cost efficiency* Massive MIMO technology is cost-effective because it uses low-power-consumption components like power amplifiers and has the potential to dramatically lower the amount of radiated power (by a factor of a thousand) [33].
- *Simple Signal Processing* Massive MIMO reduces interference effects, fast fading, and thermal noise, simplifying signal processing [34]. Massive base station antenna arrays offer crucial “channel hardening” properties. When fading channels act more predictably, the huge MIMO channel matrix gets close to the predicted values, or the count of antenna elements gets close to infinity [10].

## 4. Linear Massive MIMO

In this section, the concept of linear massive MIMO detection is discussed. Assuming a multi-user MIMO base station has *q* antennas and is serving *p* single antenna users p≤q. Assuming a frequency-flat channel, *p* users and *q* base station antennas produce a matrix (H), which may be written as follows.

$$H=\left[\begin{array}{ccc} h_{11} & \cdots & h_{1j}\\ \vdots & \ddots & \vdots \\ h_{i1} & \cdots & h_{ij} \end{array}\right],$$

where channel gain or channel impairment is given from the jth transmit antenna to the ith receive antenna. The symbol vector s can be formed after the *p* users transmit their symbols individually towards the base station, s = [s_1_,s_2_,…, s_p_]^T^.

The base station receives r = [r_1_,r_2_…,r_q_]^T^, which is corrupted by channel interference [35]. The relation between s and r can be written as follows.

(7) r=Hs+n.

The MIMO link response is expressed as a set of linear equations. The receiver will only determine the transmitted data after solving these linear equations. The stability of the solution will depend on the condition number (к(H)) of the matrix ‘H’. The condition number quantifies the two important problems in MIMO transmissions: undesirable signal correlation and noise. High к(H) is bad for the solution (ill-conditioned matrix) [36].

**Figure 5:**
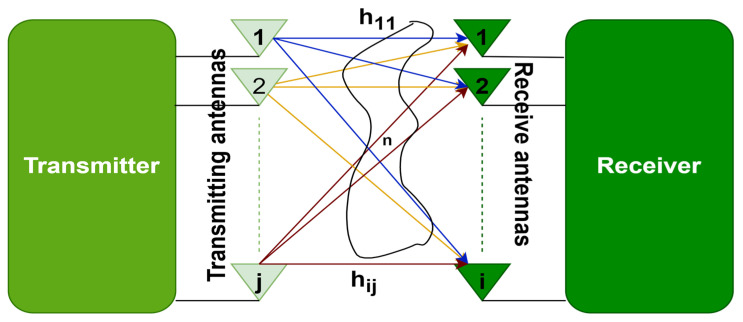
Massive MIMO with AWGN.

Massive MIMO offers high energy efficiency and spectral efficiency compared to previous technologies [33]. Figure 5 depicts the massive MIMO with additive white Gaussian noise (AWGN). The nature of high frequencies attracts the path loss effects, which will be overcome by concentrating the transmitted power in a particular narrow area by growing the number of antennas. Massive MIMO will enable us to eliminate small-scale fading, and there will be only slowly changing, large-scale fading [33]. The transmission matrix, often called channel state information (CSI), signalizes the channel condition at the time of signal transmission, signifying the good or bad behavior of the channel [37]. In SISO, the knowledge of CSI is less influential because steady-state SNR characterizes it. The CSI varies quickly in case of rapidly fading channels, and MIMO has been employed to fragment the channel variations into spatially separated sub-channels. Thus, intelligent system design is possible because of CSI knowledge.

## 5. Hybrid Beamforming and Massive MIMO

The requirement of unique RF chains for each antenna leads to an unmanageable level of complexity. In this context, suboptimal beamforming algorithms are employed that are based on a hybrid beamforming approach. Hybrid beamforming is introduced to achieve good flexibility, control over amplitude and power consumption, and cost [7]. Hybrid beamforming systems adjust digital and analog beamformers to boost the achievable rate. Hybrid beamforming was invented almost 15 years ago but has seen a dramatic upward trend over the past 5 years due to its importance in making massive MIMO cost- and energy-efficient [38]. All hybrid architectures aim to provide performance close to digital beamforming while reducing hardware and precoding complexity. The two primary types of hybrid beamforming designs are partially connected and fully connected hybrid beamforming. In partially connected hybrid beamforming, a group of antennas is coupled with an RF chain and completely connected, in which every antenna is connected to every RF chain. The two architectures pay off complexity for gain in different ways. The number of signal processing paths is (ξ × ψ^2^) for fully linked design with ξ transmit antennas and ψ RF chains, whereas it is (ξ × ψ) for sub-connected architecture. However, the fully linked architecture’s beamforming gain is ψ times bigger than the sub-connected architecture. For employing beamforming to enhance efficiency, the hybrid regularized zero-forcing (HRZF) algorithm is applied [8].

To compute the EE, a precise power model is required. This is not simple because BSs come in various forms and are typically manufactured by multiple suppliers using different execution skills. The following straightforward power model is utilized in this section:

(8) $$P_{total}={NP}_{static}+{NP}_{0}+P_{common}+{NMP}_{rf-circuit},$$

P_static_ is the base station’s static power, N_P0_ is a portion of power that varies with the number of transceivers, P_common_ is similar for any transceiver number, and MNP_rf-circuit_ scales with the total number of antennas.

*Hybrid regularized zero-forcing* (HRZF) is a precoding technique used in wireless communication with multiple antennas. It combines the regularized zero-forcing (RZF) and maximum ratio transmission methods (MRT). The HRZF precoding technique uses both RZF and MRT to achieve a trade-off between signal alignment and noise suppression while leveraging the advantages of both techniques. The RZF component helps to suppress noise and interference, while the MRT component enhances the signal power. This results in a more efficient and robust communication system and is expressed as follows.

(9) $$W=\alpha {\left(H^{H}H+\eta I\right)}^{-1}H^{H}+(1-\alpha )H^{H}{\left(HH^{H}+BI\right)}^{-1},$$

where α is the weighting factor for RZF, B is the weighting factor for MRT, and η is the regularization parameter for RZF. First, the RZF precoding matrix is estimated, and in the second step, the MRT precoding matrices are estimated; finally, the hybrid of both is applied, which is more convenient than both of its contributors.

**Figure 6:**
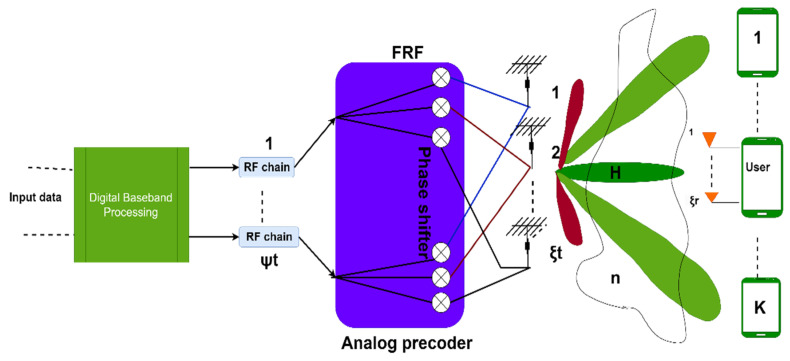
System model for hybrid beamforming in massive MIMO.

*System Model:* The system representation for hybrid beamforming in MIMO is shown in Figure 6, showing ξ_t_ number of transmitting elements and, at the receiver side, each user with ξ_r_ elements.

## 6. Results

**Figure 7:**
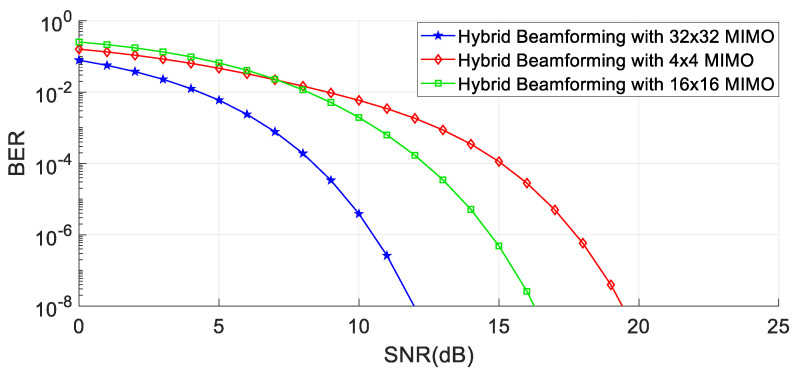
Performance with and without hybrid beamforming with different numbers of antennae.

In this section, the numerical simulation is used to show the outcomes of the proposed hybrid beamforming algorithm in conjunction with digital linear precoding methods like matched, filter, and regularized zero-forcing in the MIMO system using MATLAB software, taking into consideration the system with 28 GHz frequency, a channel sampling rate of 100 Msps, and the modulation technique used is 16 QAM. A matching number of antennas at the sender and receiver are taken into consideration. Figure 7 proves the concept of better SNR using beamforming using different numbers of antennas in the hybrid regularized zero-forcing precoding method in the MIMO system.

The maximum possible rate for each of the four receiver types, i.e., matched filter, zero-forcing, regularized zero-forcing, and hybrid regularized zero-forcing, as well as each of the channel realizations, are initialized as variables. Each channel realization results in the following.

- Production of a channel realization, which is an H-matrix with dimensions ξt × ξr.
- Calculation of each stream’s output SNR to determine the maximum possible rate for the matching filter receiver.
- Compute the maximal achievable rate for the zero-forcing receiver using the matrix inverse of H.
- Determine the maximum feasible rate for the regularized zero-forcing receiver using the matrix inverse of (H * H + LI) and a regularization parameter L, where I is the identity matrix.
- Compute the maximal achievable rate for the hybrid regularized zero-forcing receiver using two regularization parameters L_h_ and L_d,_ and the matrix inverse of (H * H + L_d_I + L_h_HH*).
- For a specific SNR value, average the highest rates that can be achieved across all channel realizations.
- Plot the maximum attainable rate against the average SNR (in dB) for each type of receiver, as shown in the below figures for different values of several antennas.

**Figure 8:**
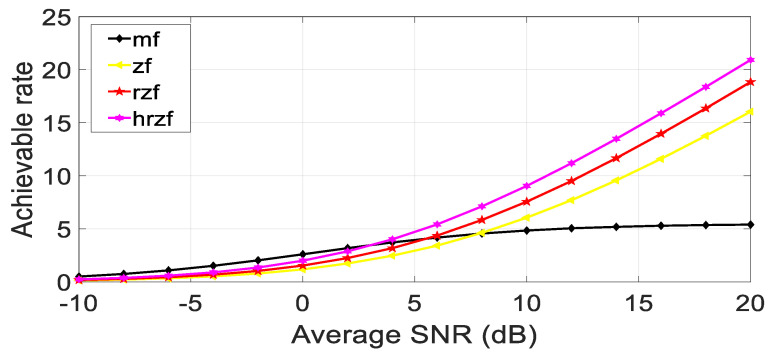
Achievable rate vs. Average SNR at ξ_t_, ξ_r_ = 4.

**Figure 9:**
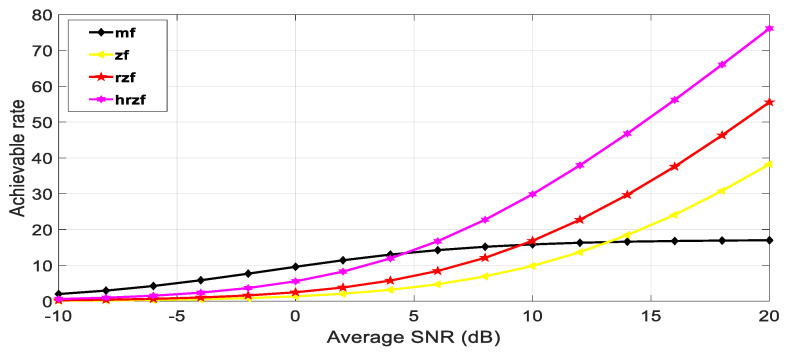
Achievable rate vs. Average SNR at ξ_t_, ξ_r_ = 16.

**Figure 10:**
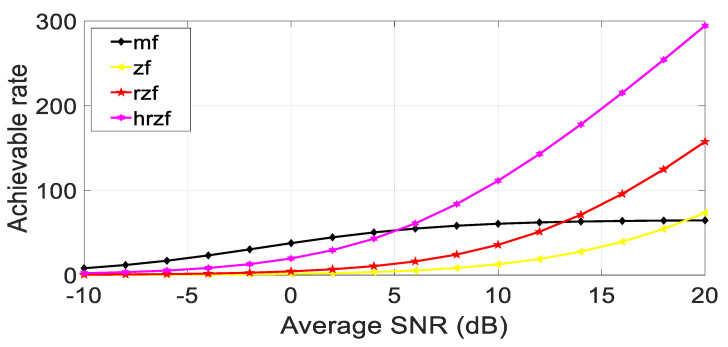
Achievable rate vs. Average SNR at ξ_t_, ξ_r_ = 64.

Meanwhile, in Figure 8, Figure 9 and Figure 10, it is quite evident that the proposed technique establishes better performance as compared to other competitor algorithms at higher SNR values (SNR > 4).

Figure 8, Figure 9 and Figure 10 show the results for 4 × 4, 16 × 16, and 64 × 64 antenna configurations. Also, the regularization parameter for channel inversion is taken as 0.05, and the regularization parameter for data covariance is 0.1 for the proposed technique. Also, it can be seen that at lower SNR values (approx. SNR < 4), the MRT technique dominated the performance curves, but as the SNR increases, its performance goes beyond the ZF and RZF as well. On comparing the results, it is observed that HRZF shows continuous growth, while other techniques show decreasing performance.

## 7. Conclusions

This paper briefly discusses the advancement in wireless telecommunications from 1G to 6G. Secondly, the concept of beamforming is introduced, and its architectural types like analog, digital, and hybrid types, along with some digital linear precoding techniques, the concept of MIMO, and its reason for introduction in wireless communication technology, is explained. Finally, the concept of hybrid beamforming in MIMO with a hybrid algorithm is described. The proposed technique shows promising results as compared to the conventional techniques used.

The configuration of the antenna array, which has been employed for the proposed beamforming, can significantly affect the performance and functionality of the system. The spatial distribution of the hybrid antenna array in the horizontal and vertical directions has further enhanced its capability to control reception signals. In the following, the software and hardware implementations of the hybrid beamforming have been fulfilled in the MathWorks Simulink visual programming environment and FPGA board, respectively. Details of the hardware implementation have been fully demonstrated on the FPGA board. The proposed optimization techniques incorporated with the hybrid antenna array have resulted in a very strong beamformer with superior capabilities and high-performance efficiency.

## Figures and Tables

**Table 1:** Investigation of beamforming in MIMO and its primary types.

References	Beamforming Technologies	Methodology	Contribution/Limitation
[4]	Introduction of beamforming with multiple antennas	Constructive interference in desired directions and destructive interference in other directions	Enhanced SNR, energy-efficient and cost-efficient
[5]	On–Off analog beamforming	A particular set of antennae is employed for beam formation	Less complex and power-saving than the analog beamforming
[6]	Introduction of digital beamforming	Every antenna element possesses an individual RF chain	More flexibility and enhanced SNR/higher complexity and higher power consumption
[7]	Hybrid beamforming	A set of antennas are connected to an RF chain	Good flexibility, comparable to digital beamforming
[2,8]	Introduction of Analog beamforming	Single radio frequency chain employed for all antenna elements in MIMO	Enhanced SNR/single beam formation at a time
[8,9,10]	Fully connected hybrid beamforming	Every RF chain is connected to every antenna element	Flexible, cost-efficient/high signal processing complexity
[10,11,12]	Partially connected hybrid beamforming	Set of antennas connected to every RF chain not overlapping each other.	Less expensive and less signal processing as compared to fully connected hybrid beamforming
[8]	Group-connected Hybrid beamforming	Antennas and RF chains are divided into η groups; signals emanating from each RF chain group are relayed via its matching antenna group	A flexible grouping strategy, a fully-connected mapping strategy is followed within each group
[13]	Hybrid beamforming in MIMO setup	Based on geometric mean decomposition-based beamforming	Large antenna gain, improved BER and SE
[4]	3D beamforming in massive MIMO	Based on user grouping and a group-based feedback system	Allows flexibility in azimuth and elevation

**Table 2:** Features and applications of 6G.

Category	Feature/Application/Challenge
Key Features	
Frequency Range	Sub-THz to THz (100 GHz to 3 THz)
Data Rate	Up to 1 Tbps
Latency	Less than 1 ms
Connection Density	10 million devices/km^2^
Energy Efficiency	10 times more efficient than 5G
Mobility	Up to 1000 km/h

**Table 3:** Analog and digital beamforming.

BF	DoF	Administration	Intricacy	Power Intake	Expenses	IUI	Information Streams
Digital	↑	ADC/DAC, Mixers	↑	↑	↑	↓	>1
Analog	↓	Phase shifters	↓	↓	↓	↑	1

## Data Availability

Not applicable.
